# Wireless sEMG System with a Microneedle-Based High-Density Electrode Array on a Flexible Substrate

**DOI:** 10.3390/s18010092

**Published:** 2017-12-30

**Authors:** Minjae Kim, Gangyong Gu, Kyoung Je Cha, Dong Sung Kim, Wan Kyun Chung

**Affiliations:** 1 Department of Mechanical Engineering, Pohang University of Science and Technology (POSTECH), 37673 Pohang, Korea; minjaekim@postech.ac.kr (M.K.); gangyong99@postech.ac.kr (G.G.); smkds@postech.ac.kr (D.S.K.); 2 Ultimate Fabrication Technology Group, Korea Institute of Industrial Technology (KITECH), 42994 Daegu, Korea; kjcha@kitech.re.kr

**Keywords:** surface electromyography, microneedle array electrode, high-density electrode array

## Abstract

Surface electromyography (sEMG) signals reflect muscle contraction and hence, can provide information regarding a user’s movement intention. High-density sEMG systems have been proposed to measure muscle activity in small areas and to estimate complex motion using spatial patterns. However, conventional systems based on wet electrodes have several limitations. For example, the electrolyte enclosed in wet electrodes restricts spatial resolution, and these conventional bulky systems limit natural movements. In this paper, a microneedle-based high-density electrode array on a circuit integrated flexible substrate for sEMG is proposed. Microneedles allow for high spatial resolution without requiring conductive substances, and flexible substrates guarantee stable skin–electrode contact. Moreover, a compact signal processing system is integrated with the electrode array. Therefore, sEMG measurements are comfortable to the user and do not interfere with the movement. The system performance was demonstrated by testing its operation and estimating motion using a Gaussian mixture model-based, simplified 2D spatial pattern.

## 1. Introduction

Muscle contraction is triggered by action potentials that propagate through muscle fibers. The combination of the action potentials can be measured from the skin surface using a technique known as surface electromyography (sEMG). This technique gathers information on movement, and it is widely used in various fields such as prosthetics [1] and rehabilitation [2]. However, the complex muscle structure makes it difficult to extract specific motion information because the sEMG signals vary depending on the electrode placement [3,4]. Thus, high-density sEMG systems [5,6,7,8] have been proposed to analyze complex muscle activity by using a spatial pattern.

A high-density sEMG system requires tiny electrodes to retrieve a high-resolution spatial distribution. Generally, wet electrodes are used for a high-density sEMG system because the conductive electrolyte enclosed in the wet electrode helps to reduce skin impedance, and assists in measuring signals from small areas by diffusing into the skin. Lapatki et al. [9] developed a thin and flexible electrode grid consisting of wet electrodes with a diameter of 1.95mm and an inter-electrode distance (i.e., center-to-center distance) of 4mm. However, wet electrodes have several limitations. An enclosed electrolyte tends to dry out and is easily contaminated, which degrades the signal quality. In addition, there might be short circuits between electrodes owing to electrolyte leakage that degrade spatial resolution, which can result in a misinterpretation. For these reasons, a wet electrode-based high-density sEMG system has limitations regarding spatial resolution and long-term use.

A solution for a high-density sEMG electrode is a microneedle array (MNA) electrode. The microneedles penetrate the high-impedance outermost skin layer and make direct contact with the more conductive living-skin layer. Therefore, an MNA electrode can measure low skin impedance sEMG signals for a long time without an additional medium. Furthermore, even a single microneedle can measure meaningful sEMG signals given its high sensitivity [10]. In other words, the electrode size of the order of micrometer can be constructed, which is an advantage in a high-density electrode configuration.

However, conventional MNA electrodes are not suitable for constructing a high-density sEMG system. Most MNA electrodes [11,12,13] have a rigid substrate, which is not suitable for variable curvature found in human skin. Rajaraman et al. [14] developed a polymer-based MNA electrode using metal transfer micromolding for simple and mass production. O’Mahony et al. [15] developed a silicon-based MNA electrode that achieves wafer level front-to-back electrical contact using through-silicon via. However, these electrodes have rigid substrates composed of the same material as microneedles. Thus, the rigid substrate hinders the stable skin contact, which can cause motion artifact and electrode displacement. Especially, in the case of MNA electrodes, the contact condition is very important because the contact area of the electrodes is small and any additional medium, which can improve contact condition, is not enclosed. Several types of flexible MNA electrodes [16,17] have been proposed for stable skin contact. Byun et al. [18] developed silicon needle arrays on the flexible polydimethysiloxane substrate. However, theses electrodes are difficult to extend to large area multi-channel configuration owing to their complex fabrication processes. For various motion estimations, electrode array should cover a large area to measure discriminative signals.

Not only the electrode specification, but also the signal processing system is important for sEMG measurement. sEMG signals are of the order of microvolts, and they are highly susceptible to noise such as power supply interference and cable artifact. Therefore, the signal amplifier unit and processing unit should be as close as possible to the electrodes for high signal-to-noise ratio (SNR). However, conventional sEMG systems [19,20,21] for high-density electrode array are bulky and require external wiring for electrode connection. These configurations are vulnerable to noise and restrict a user’s movement; hence, it is difficult to estimate a natural motion.

**Figure 1:**
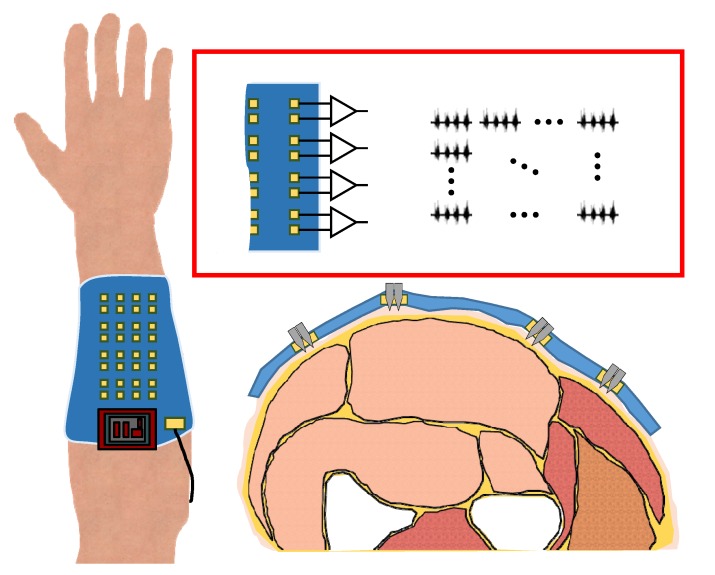
Diagram of MNHD electrode array on flexible substrate.

In this paper, a gold electroplated stainless steel microneedle-based high-density (MNHD) electrode array on a circuit integrated flexible substrate is proposed, as illustrated in Figure 1. A stainless steel needle is rigid and robust to needle breakage; thus the needle guarantees high quality signal measurement in long-term use. In addition, the needle is mass producible, and variable needle parameters such as needle body diameter and needle tip shape can be easily controlled. Furthermore, an electroplated gold layer achieves low skin-electrode impedance. The microneedle arrays were integrated to a flexible printed circuit board (FPCB). An FPCB has sufficient flexibility to cope with skin curvature, and its circuit provides stable electrical contact and robustness to external noise. The electrode parameters and size can be easily changed by designing the FPCB. Therefore, the electrode arrays can be used for various sizes of limbs according to the purpose. Measured sEMG signals from MNA electrode array are transferred to an sEMG system that consists of a signal amplifier and a processor. The system is directly connected to the FPCB without any additional wires; hence, external noise can be minimized. In the system, signal-induced features are extracted from the measured and amplified sEMG signals. Thereafter, the features can be transmitted to an external device via Wi-Fi. Therefore, the proposed wireless system provides freedom for the user’s movement and natural motion estimation is possible.

The proposed system involves signal measurement, signal amplification, feature extraction and feature transmission. In addition, Gaussian mixture model (GMM) [22]-based spatial pattern simplification is proposed for pattern-based motion estimation. Using the proposed method, all the signals are discretized by a 1D GMM, and a simplified 2D spatial pattern can be obtained. The simplified pattern can be used for not only motion estimations, but also optimal channel selections. In this study, the simplified pattern-based static finger flexion classification was conducted to demonstrate the performance of the simplified pattern-based motion estimation.

The rest of this paper is organized as follows. The fabrication of the MNHD electrode array on a flexible substrate and the sEMG system implementation are described in Section 2. The system performance is presented in Section 3. The 2D spatial pattern of the sEMG and its GMM-based simplified pattern are described in Section 4, with the corresponding motion estimation results reported in Section 5. Finally, the conclusion of the study is presented in Section 6.

## 2. Materials and Methods

**Figure 2:**
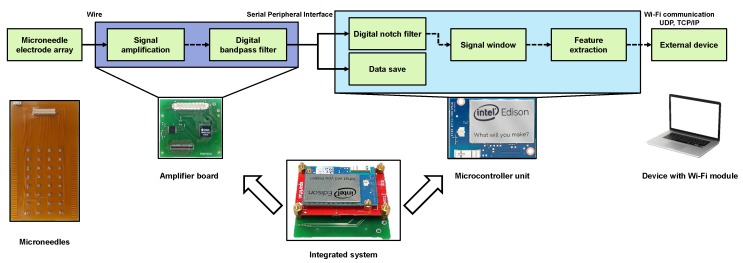
Structure of wireless compact sEMG system.

**Figure 3:**
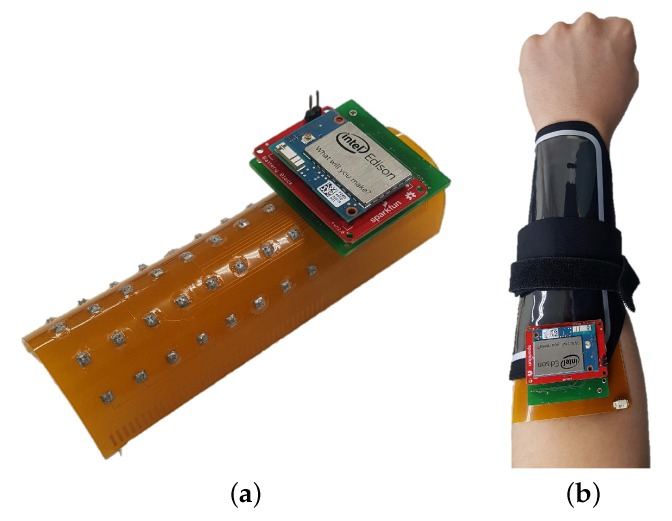
Images of (**a**) proposed system without electrode shielding, and (**b**) system attached to skin with custom band.

Both the hardware and software of the proposed wireless compact sEMG system were developed. A diagram of the complete system is shown in Figure 2, where the peripheral circuitry is connected to the MNHD electrode array to measure sEMG signals. Then, the sEMG signals are amplified, and subsequently processed and wirelessly transmitted using a microcontroller. The complete system is 16.1g and has a portable size of $44\times 42\times 11\;{\mathrm{mm}}^{3}$. The proposed system is shown in Figure 3.

### 2.1. MNHD Electrode Array

Gold electroplated stainless steel needles, having the diameter of the body as 250μm, were purchased from DASAN Engineering Co., LTD. (Gyeongbuk, Korea). To develop the MNHD electrode array on a flexible substrate, the needles were integrated in the FPCB, where the inter-electrode distance and number of needles per electrode depend on the layout.

**Figure 4:**
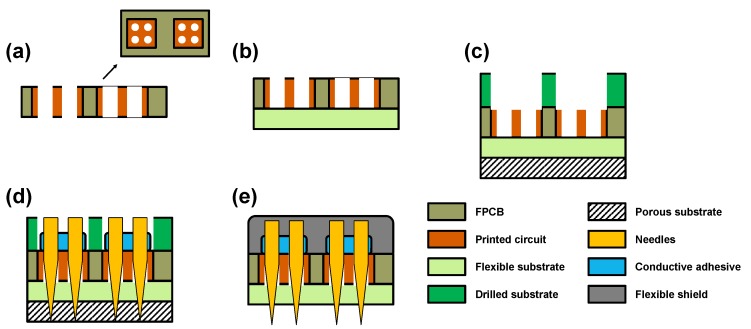
Fabrication of MNHD electrode array on flexible substrate: (**a**) patterned FPCB preparation; (**b**) soft silicone sheet attachment; (**c**) alignment of drilled substrate and porous substrate; (**d**) microneedle arrangement and fixation with conductive adhesive; and (**e**) electrode array shielding with flexible materials.

The fabrication of the array is simple and cost effective, and it is depicted in Figure 4. First, the FPCB was designed for sEMG application, as illustrated in Figure 4a. The FPCB contains holes for the microneedle array and a pad for surface-mounted components, which perform signal processing. For adequate contact with the skin and for noise minimization, an insulated soft silicone sheet was attached to the FPCB (Figure 4b). Then, a thick drilled substrate and a porous substrate were aligned (Figure 4c). The thick drilled substrate prevents the bending of the FPCB during its fabrication. Likewise, the silicone sheet secures the microneedles, and the porous substrate controls their final length during the microneedle arrangement. Moreover, the microneedle tips remain intact owing to the penetrability and softness of the porous substrate (Figure 4d). After fixating the microneedles with a conductive adhesive, flexible insulating materials were used for shielding (Figure 4e).

**Figure 5:**
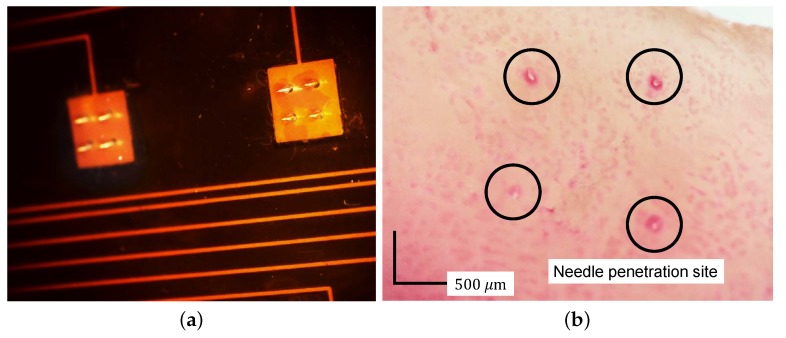
Microscopic image of (**a**) the needle array and (**b**) penetrated pig skin. Red marks indicate the needle penetration site.

The desired needle length was 200μm, at which it was determined would not cause pain. The measured needle length after the fabrication was 206μm ± 19μm (n=40), which confirmed the consistency of the fabrication as shown in Figure 5a. To demonstrate skin penetration, a pig-skin penetration test was conducted. After pressing the pig skin with the needle array, red dye was rubbed on the skin. Figure 5b shows a microscopic image of the pig skin after excess dye had been wiped off. The remaining dye marks indicate that the needle array had penetrated the skin.

**Figure 6:**
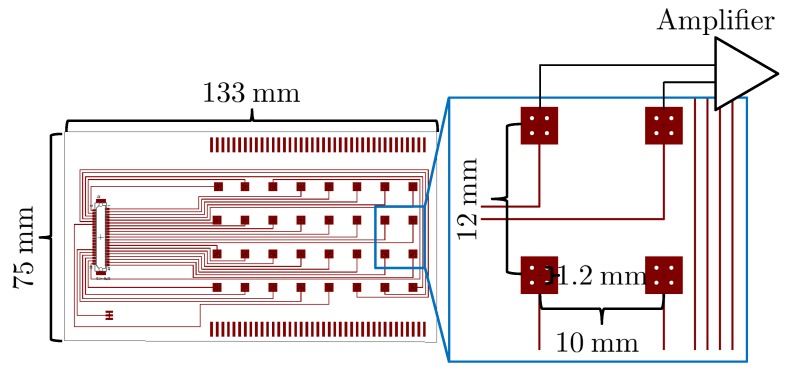
Design of FPCB used in this study.

The target limb of the developed electrode array in this study is the forearm flexor group; thus, the specific array size was designed as Figure 6 to cover the forearm flexor group. The array consists of four needles per electrode, with an inter-electrode distance of 10mm and needle-to-needle distance of 1.2mm. These parameters were determined based on previous research results [10,12]. Four needles per electrode were sufficient to measure the sEMG signals. The inter-electrode distance and needle-to-needle distance were set so that the electrode array could sufficiently cover the flexor group. The developed array contains 16 bipolar channels that are directly connected to the amplifier.

### 2.2. Signal Acquisition and Amplification

The proposed system measures sEMG signals in a bipolar configuration. The muscle activation signals measured on the four needles constituting an electrode were merged. Thereafter, the differential activation between two electrodes against a reference electrode, which is placed on the bony body part such as the elbow, was measured. Raw sEMG signals are of the order of microvolts, and are thus highly susceptible to noise sources such as power supply interference and movement artifacts. Therefore, an amplifier is required to increase the SNR. The RHD2216 amplifier (Intan Technologies LLC, Los Angeles, CA, USA) was selected. Sixteen bipolar sEMG signals were sampled at 1kHz and filtered with a bandpass filter with cutoff frequencies of 5Hz and 450Hz.

### 2.3. Signal Processing

The complexity of sEMG signals hinders their analysis. Hence, a signal processing module was embedded in the proposed system to extract and transmit useful features (e.g., the root-mean-square (RMS) value of an sEMG signal window) from such signals to an external device. For this stage, an appropriate processing unit was considered. An sEMG signal should be sampled and wirelessly transmitted every 1ms. Hence, Wi-Fi technology was selected over other transmission methods such as Bluetooth and ZigBee because it provides the required data transfer rate of 1kHz. Specifically, the Wi-Fi capability of the Intel Edison module (Intel Corporation, Santa Clara, CA, USA) that satisfies the proposed system requirements was selected. The system (including amplification, signal processing, and transmission) can operate for up to 8 h using a lithium-polymer battery of 3.7V and 400mAh.

### 2.4. Software

**Figure 7:**
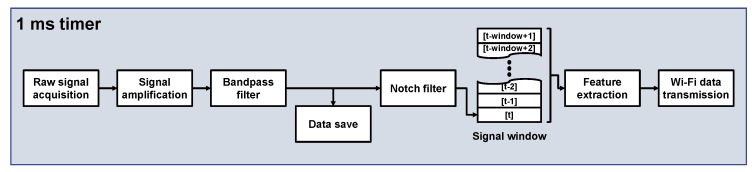
Software structure for signal processing and transmission.

The software embedded in the microcontroller processes the sEMG signal and transfers data every 1ms. The diagram of the software structure is shown in Figure 7. First, the sEMG signal is acquired by the amplifier and transmitted via a serial peripheral interface to the microcontroller. The bandpass filtered signal is then stored for further analysis. Thereafter, the signal passes through a notch filter to remove noise from the power source, and features are extracted from a predefined number of samples. The proposed system can transfer both amplified sEMG signals and their features to any external device through Wi-Fi communication. In this study, data was received by MATLAB 2017a (The MathWorks, Inc., Natick, MA, USA) using User Datagram Protocol.

## 3. System Performance

System performance can be evaluated by considering signal acquirement and processing latency. The typical bandwidth of the sEMG signal is 5–500Hz, so the sampling frequency of signal acquirement should be at least 1kHz. In addition, to construct a real-time control system, the processing delay should be minimized.

### 3.1. Signal Acquirement

The sEMG system amplifies the differential amplitude between two electrodes. Therefore, the performance can be evaluated by applying known input signals (i.e., sine waves) to the electrodes and measuring the amplifier output.

A sine wave was simulated and generated by NI PCI-6259 (National Instruments, Austin, TX, USA), and wires were connected to one channel of the system. The frequency and amplitude of the simulated sine wave were 100Hz and 1mV, respectively.

**Figure 8:**
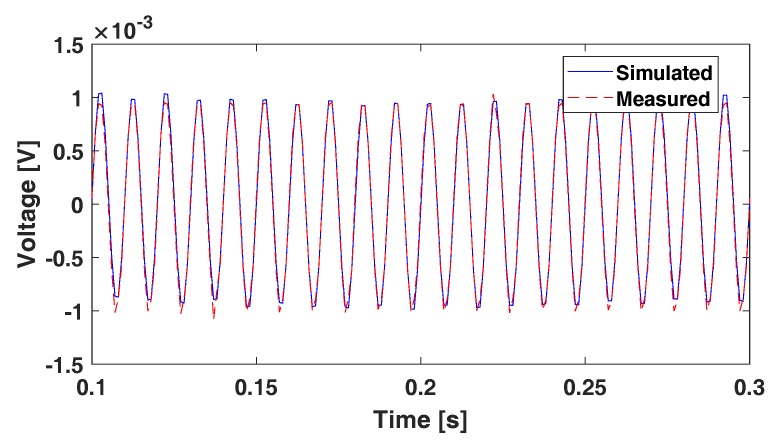
Sine wave measurement and comparison with simulated signal.

The measured signal and the simulated signal are shown in Figure 8. Although there are some peak differences between the measured signal and the simulated signal, the measured signal accurately reflected the input signal.

### 3.2. Processing Latency

During full signal processing and transmission processes (i.e., raw signal acquisition to feature transmission), the execution time and its interval loop were evaluated using the microcontroller timer and an oscilloscope. Specifically, a pulse wave was generated, and its amplitude was changed in the loop interval in which mean the frequency of the pulse wave should be 500Hz. The processing and transmitting execution time was measured using the microcontroller built-in function, and the pulse wave was measured using the oscilloscope.

**Figure 9:**
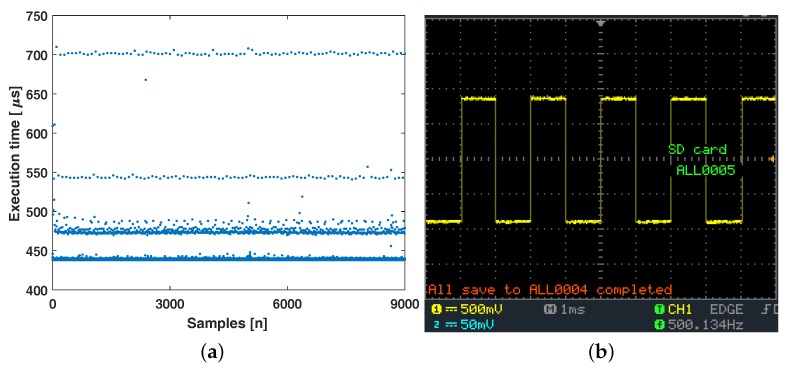
Processing latency evaluation: (**a**) processing and transmission execution time evaluation during 9s of measurement; and (**b**) loop interval measurement using oscilloscope.

The experimental results are shown in Figure 9. A total of 9s of execution time of each loop was measured. Generally, the execution took less than 500μs, and the longest execution time was less than 750μs, as shown in Figure 9a. The measured frequency of the pulse wave was almost the same as the ideal frequency value of 500Hz, as shown in Figure 9b. The result indicates that the overall processes can be performed within 1ms and that the system maintains a constant 1kHz processing and transmitting frequency.

## 4. Pattern-Based sEMG Signal Analysis

**Figure 10:**
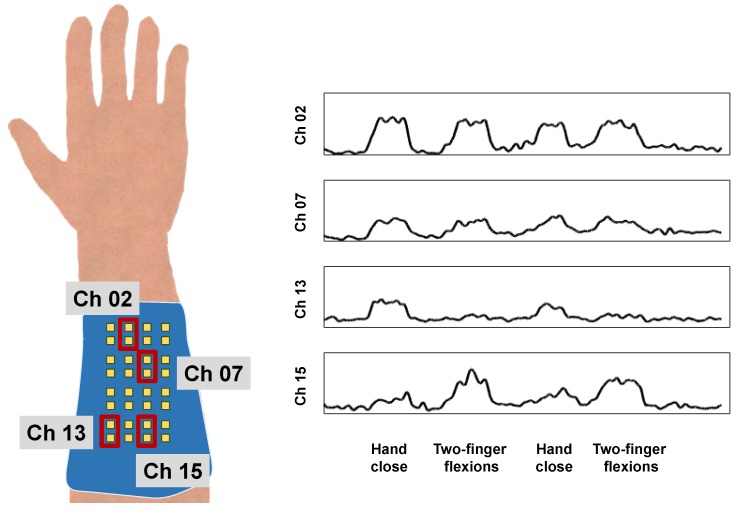
Schematic image of channel distribution and measured signals from specific channels during several muscle contractions.

The measured sEMG signals vary depending on the electrode placement. Hence, spatial patterns retrieved from different electrodes in a specific area can be used to retrieve motion information. As an example, the sEMG signal during hand-close and two-finger (index finger and middle finger) flexions were measured. The electrode array was placed on the flexor group of the left forearm and the reference electrode was place on the elbow. The motions were performed sequentially. Then, the measured signals were divided into 200ms windows every 5ms, and the RMS value of each window was extracted as a feature. The moving-average filtered RMS signals are shown in Figure 10.

During the hand-close motion, channel 2 exhibited higher amplitudes than channels 7, 13, and 15. During two-finger flexions, channels 2 and 7 showed a similar tendency in that the exhibited amplitudes were similar to the amplitudes during the hand-close for each channel. On the other hand, channel 13 exhibited a weak amplitude compared with the amplitude during the hand-close. By contrast, channel 15 exhibited a higher amplitude than that during the hand-close. Thus, the sEMG activation signal amplitude varies according to motion, which is affected by the electrode placement.

**Figure 11:**
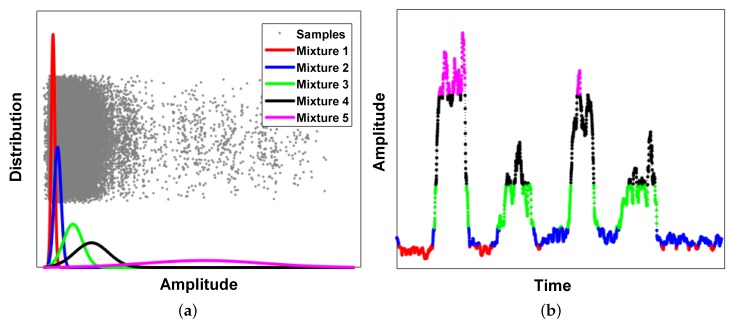
Amplitude clustering using GMM: (**a**) constructed GMM using all samples from channels; and (**b**) clustered amplitudes of signal from channel 9.

To obtain a simplified spatial pattern, signals were discretized by a GMM, which is a clustering method. The model retrieved the probabilistic model parameters from a set of samples. One-dimensional GMM was trained using RMS signals from all channels. Figure 11 shows the cluster of signals from channel 9 using five Gaussian mixtures. Figure 11a shows the amplitude distribution from all channels and the constructed GMM. Figure 11b shows the RMS signal from channel 9 and its indexed cluster according to the constructed GMM.

**Figure 12:**
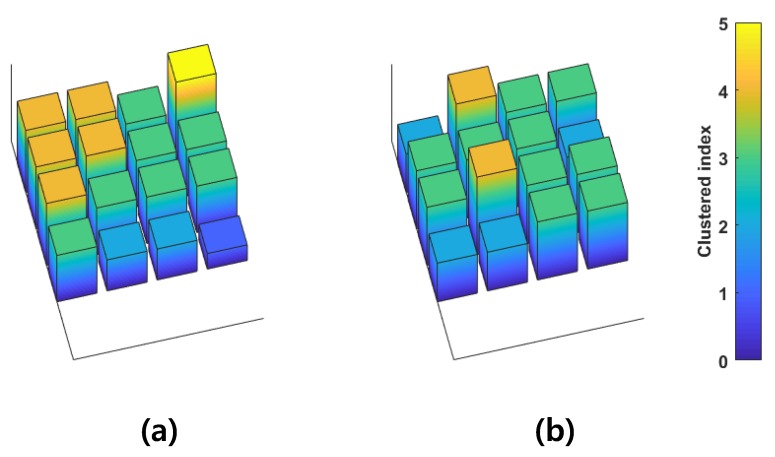
GMM-based retrieved 2D spatial pattern during (**a**) hand-close and (**b**) two-finger flexions.

The simplified 2D spatial patterns during hand-close and two-finger flexion are shown in Figure 12. The different activation patterns were observed and can be used to retrieve motion information. The number of Gaussian mixtures may affect the discrimination performance, and there may be trade-offs. However, in this case, even two Gaussian mixtures were enough to discriminate the motions (not described here).

## 5. Motion Estimation

In this section, finger-flexion discrimination was conducted to demonstrate the performance of the system and GMM-based simplified 2D spatial pattern. The finger-motion-related muscles in the forearm have a complex structure. For example, flexor digitorum superficialis has four tendons connected to individual fingers. A 2D spatial pattern can provide meaningful motion information owing to its high spatial resolution.

**Figure 13:**
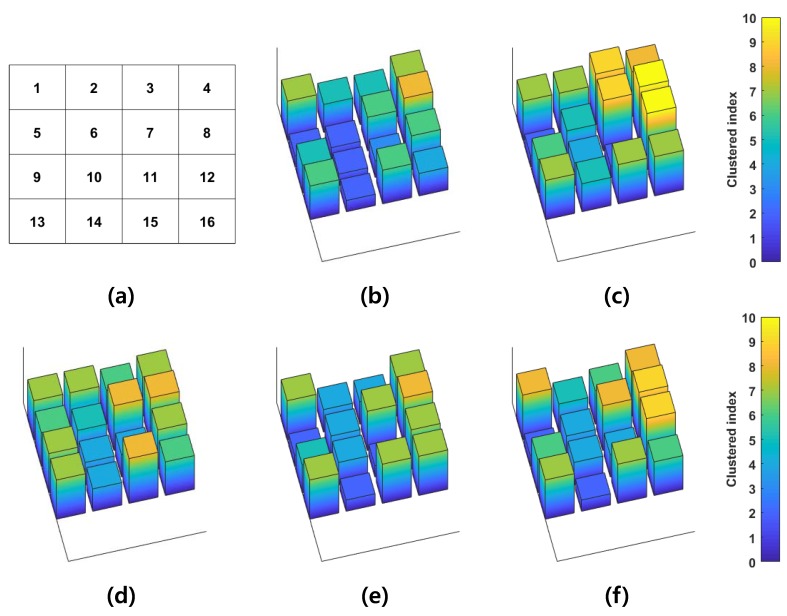
GMM-based retrieved 2D spatial pattern during finger flexion: (**a**) channel distribution; (**b**) thumb finger flexion; (**c**) index finger flexion; (**d**) middle finger flexion; (**e**) ring finger flexion; and (**f**) little finger flexion.

**Figure 14:**
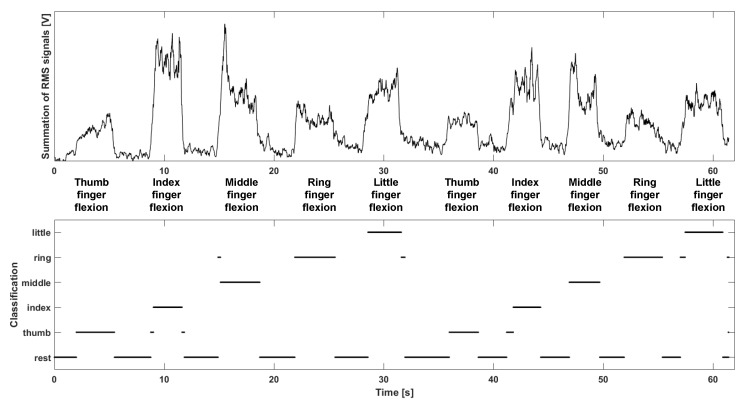
Summation of RMS signals from all channels, and finger flexion classification.

The system was placed on the flexor group of the left forearm. Signals were measured during single-finger flexion motions (thumb, index, middle, ring, and little finger). Then, the measured signals were divided into 200ms every 5ms, and the RMS value of each window was extracted. One-dimensional GMM was constructed by signals from all channels. Simplified 2D spatial patterns of each motion are shown in Figure 13, where the number of Gaussian mixtures is 10. Channels 1, 4, and 8 are commonly activated during finger flexions. On the other hand, several channels were selectively activated according to motion. For example, channel 5 was activated only during middle finger flexions. The relationship between the simplified 2D spatial pattern and motion was estimated via a neural network classification by utilizing the Neural Network Toolbox software package in MATLAB 2017a. Then majority vote [23] was applied. The classification result is shown in Figure 14.

These patterns were highly related to the anatomical parameters of the muscle and electrode array placement. Thus, 2D spatial pattern has high inter-subject variability and may not guarantee the performance even on a single subject when the electrode array placement is changed. In a future study, we plan to develop a muscle model-based electrode array placement recognition for robust motion identification.

## 6. Conclusions

This paper reports on a gold electroplated stainless steel microneedle-based high-density (MNHD) electrode array on a circuit integrated flexible substrate with a compact wireless system for surface electromyography (sEMG). A stainless steel needle is rigid and robust to needle breakage; thus, the needle guarantees high-quality signal measurement in long-term use. In addition, the needle is mass producible and, variable needle parameters such as needle body diameter and needle tip shape can be easily controlled. Furthermore, an electroplated gold layer achieves a low skin-electrode impedance. The flexible substrate has sufficient flexibility to cope with skin curvature, and its circuit provides stable electrical contact and robustness to external noise. The wireless signal processing system transmits the features to an external device via Wi-Fi. Therefore, the proposed wireless system provides freedom for the user’s movement, and a natural motion estimation is available. Additionally, simple and effective GMM-based simplified pattern is proposed for motion estimation.

The performance of the system was evaluated by considering signal acquirement and processing latency. In addition, finger motion recognition was demonstrated to prove the simplified pattern-based approach. The experimental results indicated that the proposed system can measure and transmit reliable sEMG signals in real time, and the GMM-based simplified pattern can be used to estimate motions.

## Abbreviations

The following abbreviations are used in this manuscript:

sEMG	Surface electromyography
SNR	Signal-to-noise ratio
FPCB	Flexible printed circuit board
MNA	Microneedle array
MNHD	Microneedle-based high-density
RMS	Root-mean-square
GMM	Gaussian mixture model
